# Green synthesis of multifunctional carbon coated copper oxide nanosheets and their photocatalytic and antibacterial activities

**DOI:** 10.1038/s41598-021-90207-5

**Published:** 2021-05-24

**Authors:** Hamida Bibi, Mudassar Iqbal, Hassan Wahab, Mehmet Öztürk, Fei Ke, Zafar Iqbal, Muhammad Ishfaq Khan, Suliman Mohammed Alghanem

**Affiliations:** 1 Department of Soil and Environmental Sciences, The University of Agriculture, Peshawar, Pakistan; 2 Department of Agricultural Chemistry and Biochemistry, The University of Agriculture, Peshawar, Pakistan; 3 Physics Division, Pakistan Institute of Nuclear Science and Technology, Nilore, Islamabad, Pakistan; 4 Department of Chemistry, Faculty of Sciences, Muğla Sıtkı Koçman University, Muğla, Turkey; 5 Department of Applied Chemistry and State Key Laboratory of Tea Plant Biology and Utilization, Anhui Agricultural University, Hefei, People’s Republic of China; 6 Department of Weed Science and Botany, The University of Agriculture, Peshawar, Pakistan; 7 Biology Department, Faculty of Science, Tabuk University, Tabuk, Saudi Arabia

**Keywords:** Environmental sciences, Natural hazards, Chemistry, Materials science, Nanoscience and technology

## Abstract

The studies of metal oxides in environmental remediation of chemical and biological pollutants are gaining colossal importance. Herein, we report the facile synthesis of multifunctional copper oxide nanosheets (CuO NS) using an aqueous extract of *Rhazya stricta.* The phytochemical investigation of *R. stricta* indicated the presence of saponins, tannins, and reducing sugars, responsible for the reduction and stabilization of CuO NS. A UV–Visible spectrophotometer initially confirmed the fabrication of CuO NS with specific Surface Plasmon Resonance at 294 nm. Field Emission Scanning Electron Microscopy (FE-SEM), Fourier-transform infrared spectroscopy FTIR, and XRD were further used to characterize the CuO NS. The obtained CuO NS were poly-dispersed with an average size of 20 nm. Interestingly these particles were aligned together in 3D cubical sheets layered above each other via self-assembly. The as-synthesized CuO NS showed enhanced antibacterial potential (17.63 mm, overall mean inhibition zone) in comparison to the known antibiotics (11.51 mm, overall mean inhibition zone) against both *Solanaceous* crop's wilt-causing bacteria (*Ralstonia solanacearum* and *Clavibacter michiganensis*). Furthermore, the appreciable photocatalytic potential of CuO NS has also been observed, causing 83% degradation of methylene blue (MB) upon solar irradiation. The synthesis methodology is devoid of any toxic waste or by-products. It could be used to produce eco-friendly CuO nanomaterial for industrial uses.

## Introduction

Metal oxide nanostructures have drawn considerable interest due to their enhanced photocatalytic properties, low cost and wide range of biological and industrial applications^1,2^. Besides having excellent antimicrobial properties, metal oxide nanoparticles could be used for drug delivery and against multidrug resistance (MDR) pathogens. Metal nanostructures such as silver (Ag), gold (Au), and Iron (Fe) have been widely studied for their bioactivity and applications in various consumer products^3–5^. These can be synthesized using different methods, including chemical^6^, electrochemical^7^, sol–gel^8^ and condensation^9^, but with certain limitations such as the generation of hazardous waste, use of toxic chemicals and solvents, the difficulty in optimizing the extent of scaling up synthetic processes, and utilization of high energy^10^. The recent development in nanotechnology combined with green chemistry led to the development of environmentally friendly, non-toxic, and cost effective procedures for the fabrication of nanomaterials^11^. Due to the large surface to volume ratio, the nanomaterials are highly reactive and results in extremely beneficial properties, including mechanical, biochemical, biotechnology, optics, catalysis, and medicines^12^.

Recently copper oxide nanostructures have gained attention due to their low cost compared to the existing metal nanoparticles such as Ag and Au. They are also considered almost ten times cheaper than their other counterparts. Copper oxide nanostructures are believed to have high sensitivity against Gram-negative & Gram-positive microorganisms. They have a high potential as an external microbial agent and a biocidal film over medical devices^13^. However, minimal studies are available for their use in agriculture to control pathogens.

Wilting of solanaceous crops caused by bacteria, including *Ralstonia solanacearum* and *Clavibacter michiganensis,* is of significant concern for the growers^14^. Both these bacteria spread rapidly on their outbreak and cause severe damage by clogging the vascular system through extracellular polysaccharides^15,16^. These pathogenic bacteria are generally controlled using commercially available antibiotics that are expensive and pose threats to the environment. Furthermore, rapid industrialization and excess use of dyes pose a severe threat to the water quality and biotic ecology. These organic pollutants are highly mutagenic and carcinogenic to human health. Dyes are poorly degradable, and their complex chemical structure makes them difficult to remove from water^17^. Therefore, it is of utmost importance to have an eco-friendly products for the control of these pathogens and for the removal of both biological and chemical pollutants.

To the best of our knowledge, there has been no report on the single-step synthesis of carbon-coated CuO-NS using an aqueous extract of *R. stricta*. Herein, we report a one-pot, environmentally friendly method for the fabrication of multifunctional CuO NS for photocatalytic degradation of methylene blue (MB) and their possible use as a bio-control agent against wilt causing bacterial pathogens of solanaceous crops. This research will ultimately open new avenues in the agriculture and environment sectors.

## Materials and methods

### Collection of plant material

**Figure 1:**
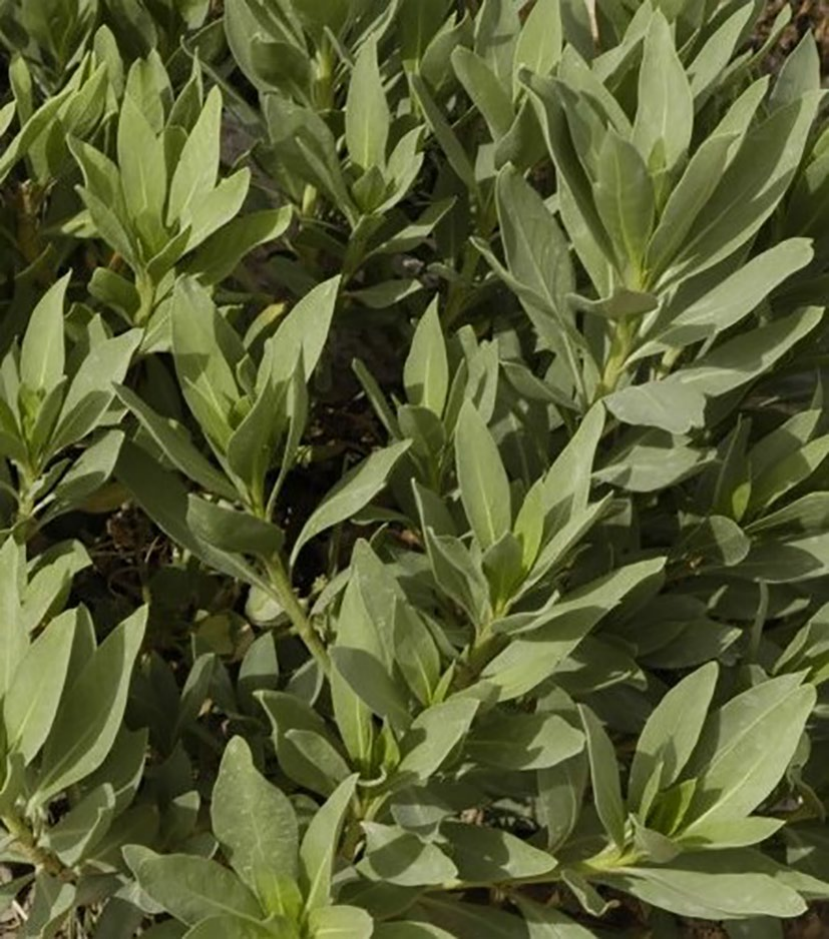
Photo of *Rhazya stricta* grown in natural habitat.

The collection of plant material was carried out according to the relevant national and international guidelines^18^. Arial parts of *Rhazya stricta* (Wild) were collected with permission from local forest officers in the month of April 2020 from district Karak, Khyber Pakhtunkhwa, Pakistan, located at 33°7′12 N 71°5′41E (Fig. 1). The plant samples were stored in a paper bag and transferred to the laboratory within 48 h and identified by a botanist. A specimen of the sample was deposited in a special herbarium for weeds and medicinal plants (voucher No. MIK-5/20-397), Department of weed science and botany, The University of Agriculture Peshawar. The collected samples were rinsed with distilled water and air-dried under shade for 14 days. The dried samples were chopped into small pieces using a sterile scissor and heated in distilled water (10% weight to volume ratio) at 60 °C for 20 min on the heating mantle. The solution was cooled to room temperature, vacuum filtered to obtain the aqueous extract concentrate (brown color), and stored at 4 °C for further use.

### Qualitative analysis of phytochemicals

The phytochemical contents of *Rhazya stricta,* including alkaloid, phenols, flavonoids, terpenoid, saponins, steroids, tannins, and anthraquinones, were assessed using standard procedures^19,20^.

### Biosynthesis of CuO NS

For the synthesis of CuO NS, 60 mL of freshly prepared aqueous extract of *Rhazya stricta* was added to 80 mL of 0.5 M aqueous copper sulfate pentahydrate (CuSO_4_.5H_2_O) solution at room temperature. A noticeable change in color from blue to green was observed as soon as both solutions come in contact, indicating the reduction of ionic copper. Upon heating at 80 °C for 12 h, the green solution turned brown. The reaction mixture was then allowed to cool at room temperature, and the brown suspension was collected through centrifugation (10,000 RPM for 10 min). The precipitates were thoroughly washed with distilled water and absolute ethanol to remove unreacted reagents and biomolecules. The obtained solid material was dried under N_2_ flow and stored at 4 °C for further analysis.

### Characterization copper oxide nanosheets

#### UV–Visible spectral analysis

The bio-reduction of Cu^2+^ ion solution through an aqueous extract of *Rhazya stricta* was monitored using a split-beam UV–Vis spectrometer (Optima Sp3000 + Japan) for its maximum absorption v/s wavelength range against the aqueous extract of *Rhazya stricta* as a blank. Upon completion, the reaction mixture was centrifuged at 10,000 rpm for 10 min to eliminate any uncoordinated bio-molecules. The obtained solid material was re-suspended in double-distilled de-ionized water and scanned from 200–1100 nm wavelength.

#### Fourier transform infrared spectroscopy (FT-IR) analysis

The Fourier Transform Infrared spectra were recorded under identical conditions in the range 400–4000 cm^−1^ region using FTIR Spectrometer (SHIMADZHU; Japan).

#### X-ray diffraction analysis

The phase identity, crystalline structure, and crystallite size were determined from the XRD data using Cu-kα radiation source. The CuO NS powder was coated on a glass substrate and submitted for their crystal structure analysis. The results were recorded as a graph with 2θ vs. intensity at the x-axis and y-axis, respectively.

#### Field emission scanning electron microscopy (FE-SEM)

The surface morphology and size distribution of as-synthesized CuO NS were characterized using TESCAN MAIA3 Field Emission Scanning Electron Microscopy (FE-SEM). A drop of an aqueous Cu NS solution obtained after purification via repeated centrifugation was placed on a Silicon (Si) substrate and let to dry and characterized at an accelerated voltage of 5.0 kV.

#### Antibacterial assays

Synthesized CuO NS were tested for inhibition against *Solanaceous* crops' wilt-causing bacterial pathogens, including *Ralstonia solanacearum* and *Clavibacter michiganensis*. The antibacterial assay was carried out by the disc diffusion method^21^. Both bacterial strains were developed using nutrient broth at 38 °C for 24 h and then streaked over Potato Dextrose Agar's (PDA) surface using sterile cotton swabs. The sterile paper disk (5 mm) was adsorbed with 10 µL of the reaction mixture (6.23% in sterile water) and placed on the PDA surface. As a control 10 µL plant extract (10.00%), 10 µL 0.5 M aqueous CuSO_4_ solution (6.23%), and a positive control 10 µL of 6.23% streptomycin (0.1 M in distilled water) were separately adsorbed on sterile paper discs (5 mm). All the prepared disks were placed on a prepared lawn of bacterial cultures on PDA to assess their effect on pathogens. The plates were incubated at 38 °C for 24 h, and bacterial growth inhibition was observed as clear halos (zones) around the discs.

### Photocatalytic property

The photocatalytic potential of biogenic CuO NS was carried out at the end of May under sunlight irradiation. To 100 mL (10 µg mL^−1^) aqueous methylene blue (MB) solution in a transparent flask, 10 mg of synthesized CuO NS were added and allowed to stir for 2 h and 20 min under direct sunlight. A control experiment (in the absence of CuO NS) was also maintained simultaneously. After every 20 min of reaction duration, 5 mL sample aliquot was collected and centrifuged (10,000 rpm) for 6 min to remove suspended CuO NS. The supernatant was collected and examined by a UV–Vis spectrophotometer at λ_max_ 664 nm to estimate available unreacted MB. The following formula was used to determine the percent photodegradation efficiency (PD%) of MB.

$$PD\%= \frac{{\mathrm{C}}_{0}-{\mathrm{C}}_{t}}{{\mathrm{C}}_{0}}\mathrm{X}100$$

where PD% denote percent photodegradation, C_0_ and C_t_ represents the absorbance of MB at time 0 min and t min.

### Statistical analysis

The data was obtained in triplicates. The antibacterial activity is presented as a mean zone of inhibition (mm) ± S.E, the different letters in columns are statistically significant at *p* < 0.05.

## Results and discussion

The detection of phytochemical in aqueous extract of *Rhazya stricta* revealed the presence of various groups of bioactive natural products (Table 1). These natural products, especially polyphenols, are believed to reduce metal ions and the formation of metallic nanoparticles^22^.

**Table 1:** Phytochemical analysis of *Rhazya stricta*, + shows the presence and − shows the absence of molecules.

	Saponins	Alkaloids	Tannins	Flavonoids	Polyphenols	Anthraquinones	Steroids
*Rhazya stricta* aqueous extract	+	+	+	+	+	−	−

### Biosynthesis of CuO NS

The synthetic procedure for CuO NS using an aqueous extract of *Rhazya stricta* is very simple, environmentally friendly, and cost-effective. Here the CuSO_4_ dissolved in water dissociates into Cu^2+^ and SO_4_^2−^ ions. The Cu^2+^ is then reduced to CuO by the action of biomolecules (predominantly polyphenols) present in the aqueous extract of *Rhazya stricta*. The generated metallic copper nuclei then start growing into nanosheets. Several biomolecules from *Rhazya stricta* distributed in a reaction then stabilized CuO NS. This copper NS then starts precipitating due to water insolubility. The unreacted biomolecules sticking the surface of nanoparticles were removed during washing with distilled water and ethanol, resulting in pure dark brown CuO nanosheets powder.

### Characterization of CuO NS

#### UV–Vis spectroscopy analysis

**Figure 2:**
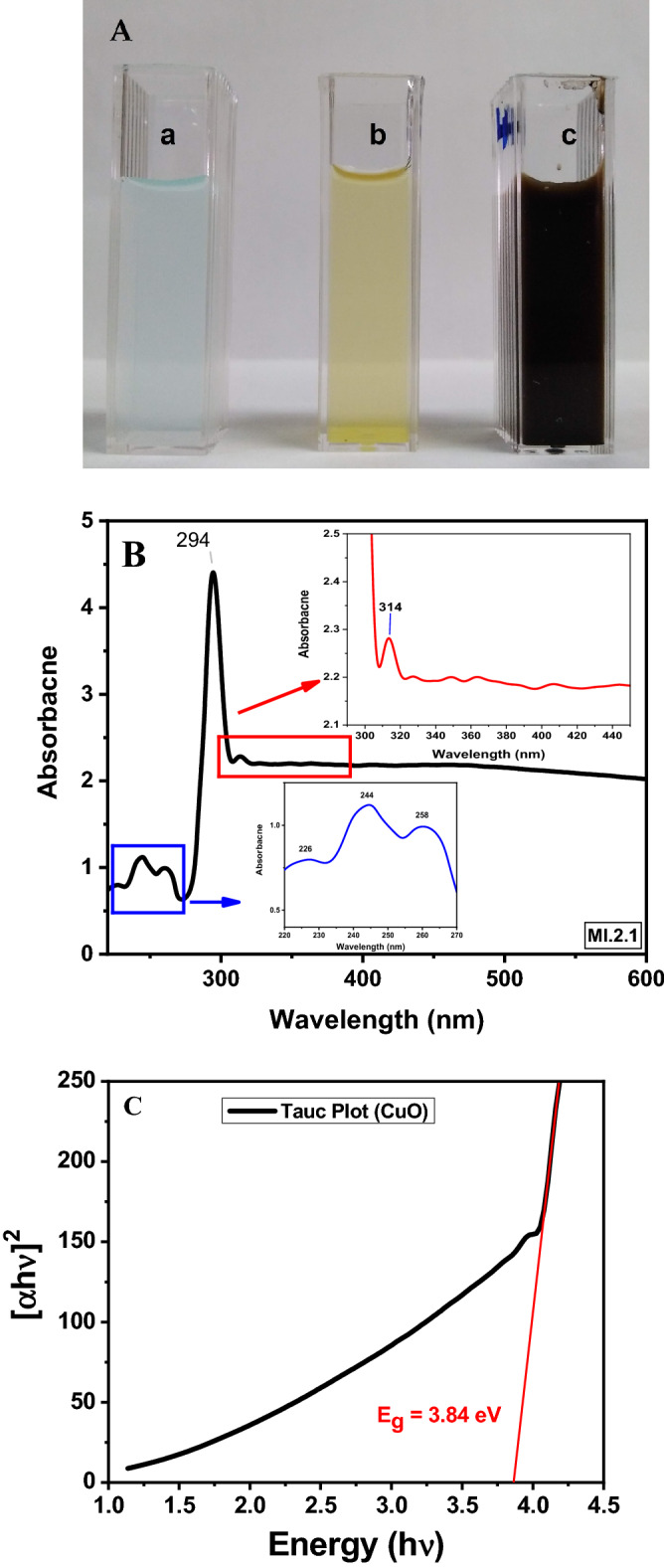
(**A**) Photograph of sample changing color (a) aqueous CuSO_4_ solution, (b) aqueous extract of *Rhazya stricta*, (c) aqueous solution of CuO NS. (**B**) UV visible absorption spectrum of CuO nanosheets. (**C**) Tauc’s plot showing the energy bandgap of CuO NS. Both absorption spectrum (**B**) and Tauc's plot (**C**) were produced by OriginPro 2018 (64bit).

The addition of aqueous extract of *Rhazya stricta* to the CuSO_4_ solution led to a change in the color from blue to green which turned brown after heating at 80 °C, indicating the formation of CuO NS's (Fig. 2A). This change in the color is due to the excitation of surface plasmon vibration of CuO NS, which generated a high-intensity peak appearing at 294 nm. A minor peak at 314 could be from the different sizes/shapes of CuO NS (Fig. 2B Inset)^23^. We opined that this is also attributed to the n → π* transition of C=O bonds^24^. The peak appearing at 226 nm, 244 nm and 258 nm (Fig. 2-B inset) in the low-frequency region are the characteristic peaks of graphene nanosheets corresponding to the π conjugation network or π → π* transition^25^. The presence of low-frequency peaks (Fig. 2B-inset) confirms that the biomolecules present in the aqueous extract of *Rhazya stricta* might have played a role in reducing and stabilizing CuO NS. Amorphous carbon has a less sharp peak and an extended band tail than graphitic carbon^26^. The peak positions of onion-like nano carbon depended on the shell structure. For example, the spherical, polyhedral, and ribbon-like onion structures had peaks at 233, 241, and 249 nm, respectively^27^. Tan and co-workers^28^ have attributed the peak at 220 nm to a disordered and defective carbon. The high-frequency peaks might be attributed to the existence of nanoribbon like carbon sheets surrounding the CuO NS.

The bandgap of CuO NS was determined using the Tauc plot method^29^, by plotting (αhν)^2^ versus ℎν, as shown in Fig. 2C, where α is the absorption coefficient, *hv* is the energy of incident light, *h* is Planck's constant (6.626 × 10^−34^ Js), and *v* is the frequency of light. Based on tauc's plot, the bandgap was calculated as 3.84 eV. A similar energy band gap (3.85 eV) was reported by Dhineshbabu and co-workers while studying the structural and optical properties of cupric oxide nanoparticles synthesized via sonochemical method^30^. More recently, a slightly higher bandgap of 4.01 eV of CuO nanoparticles has been reported using melanin as a stabilizer agent^31^.

#### X-ray diffraction analysis

**Figure 3:**
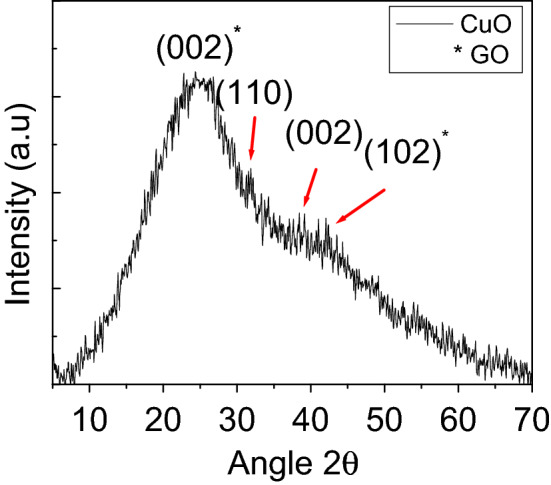
XRD spectrum of CuO NS synthesized via green synthesis using *Rhazya stricta* aqueous extract showing major diffraction peaks. XRD spectrum was produced by OriginPro 2018 (64bit).

Figure 3 shows the X-ray diffraction (XRD) pattern of the carbon-coated CuO NS. Overall the spectrum shows an amorphous nature of the CuO NS. However, the appearance of some small diffraction peak at 2θ = 23.8°, 2θ = 31.90°, 2θ = 38.52° and a weak shoulder at 2θ = 41.22°, confirms the growth of crystals and formation of some crystalline planes. The peaks appearing at 2θ = 23.8° and 2θ = 41.22° are believed to be due to the emergence of (002) and (102) respectively for graphene oxide, and the peaks appearing at 2θ = 31.90°, 2θ = 38.52° are attributed to the (110) and (002) planes for CuO^32,33^. The lattice spacing determined from the (002)* plane is of the order of 0.37 nm, while for crystalline graphite, the lattice spacing remains about 0.34 nm. The slightly higher d spacing value suggests the presence of some oxygen functional groups between the carbon layers. Besides this, the broadened peak corresponding to the (002)* plane is believed to be due to the disordered carbon structure indicating the formation of graphene sheets as observed by Ashish and Sundara^34^, which might be due to the self-assembly of carbon atoms.

#### Fourier transform infrared spectroscopy (FT-IR) analysis

**Figure 4:**
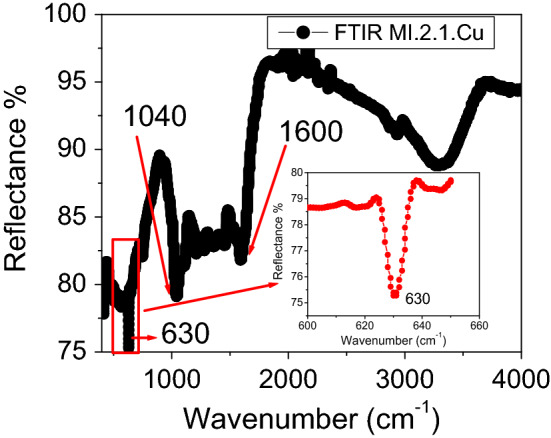
FTIR spectrum showing various absorption bands. The inset magnified part of the spectrum showing clearly the sharp peak at 630 cm^−1^ corresponding to the formation of pure CuO nanosheets. OriginPro 2018 (64bit) was used to plot data.

Various bio-molecules having different functional groups were responsible for reducing CuSO_4_ and then stabilizing CuO NS derived from bioactive molecules. Various functional groups were found to be attached to nanosheets' surface during synthesis. FTIR is one of the most important techniques used to identify functional groups attached to nanomaterials' surface. Figure 4 shows a sharp band appearing at 630 cm^−1^, the characteristics band of pure monoclinic CuO, as reported earlier^35^. The peak appearing at 1040 cm^−1^ is attributed to the C-H bending vibrations. A sharp absorption band at 1600 cm^−1^ is believed to be due to the self-assembled disordered carbon sheets. The broad peak at 3297 cm^−1^ is believed to be due to OH groups' existence on the surface of CuO NS. A sharp absorption band at 2930 cm^−1^ is because of the CH and CH_2_ groups. The appearance of bands at 1600 cm^−1^ and that of the 2930 cm^−1^ is believed to predict graphene sheets interacting with CuO NS^36^. Chemical linkages on the surface of CuO NS suggest that the hydroxyl and carbonyl groups might have reacted as reducing and stabilizing agents for the fabrication of CuO material and, consequently, accumulating CuO NS.

#### Field emission electron microscopy of CuO NS

**Figure 5:**
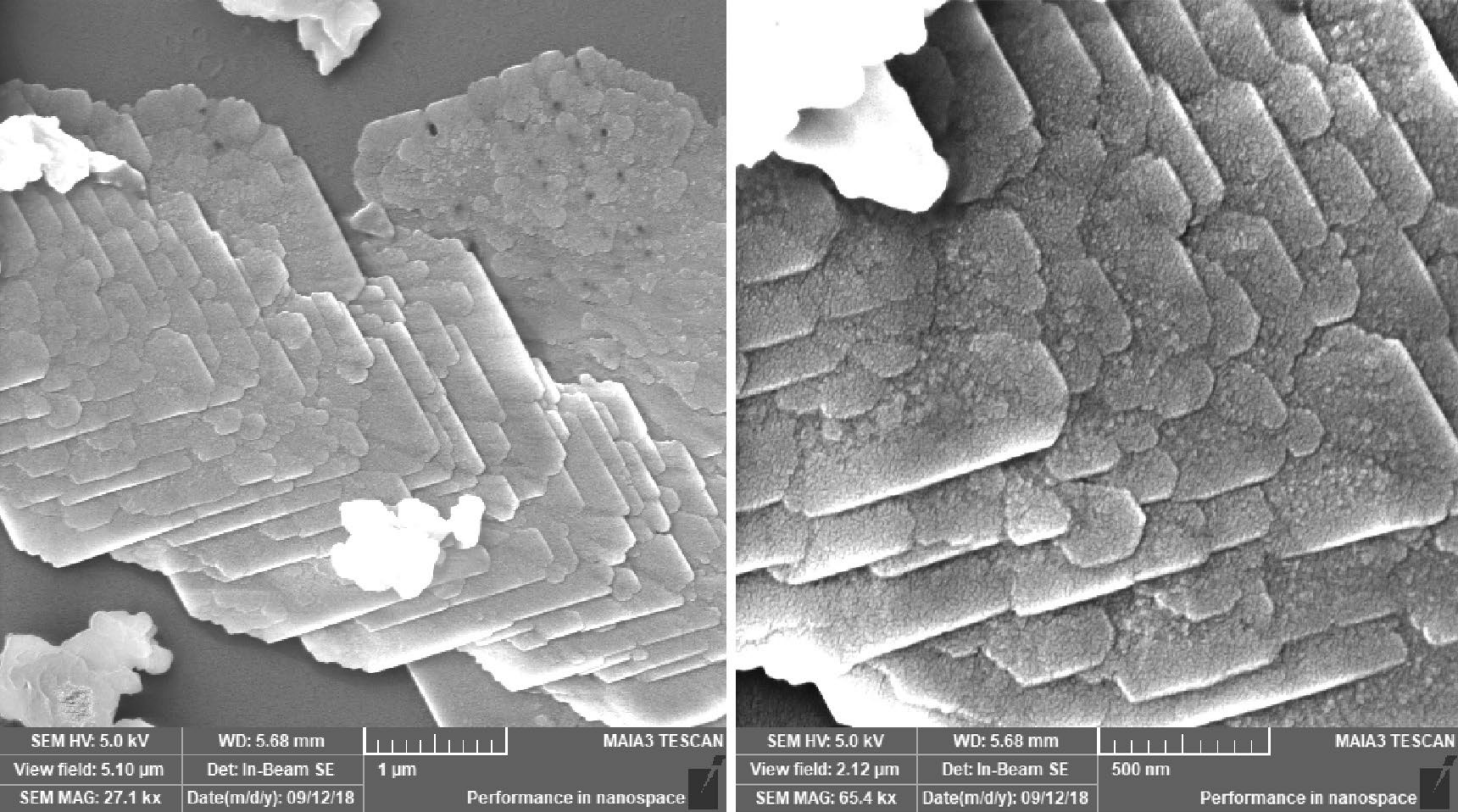
FESEM image as-formed Copper Oxide nanosheets.

The field emission scanning electron microscopy (FE-SEM) was used to analyze the morphology of as-prepared CuO nanosheets, as shown in Fig. 5. It can be observed that CuO crystallizes in the form of smaller nanoparticles, which are polydispersed and aligned together in the proper sequence. These nanoparticles, upon diffusion, lead to the formation of nanosheets. The sheet's width appears to be less than 20 nm, which leads to highly reactive edges and corners.

#### Antibacterial potential of CuO NS against wilt causing pathogens

The antibacterial properties of CuO NS were evaluated against the wilt-causing bacterial pathogens of *Solanaceous* crops. Two bacterial strains, including *Clavibacter michiganensis* (Gram-positive) and *Ralstonia solanacearum* (Gram-negative), were tested using the disk diffusion method. The synthesized CuO NS showed significant inhibition zones against bacterial strains. The Cu ions are known to disrupt various biochemical processes^37^. The bacterial cell wall comprises both amine and carboxyl groups^38^, which shows a high affinity towards the copper ions^39^. The CuO having a large surface to volume ratio may also bind with the nucleic acid of bacteria destroying the helical structure of DNA. The cationic Cu released from nanoparticles can attach to the negatively charged portion of the bacterial cell wall resulting in denaturation of protein and subsequently causing cell death^40^.

**Figure 6:**
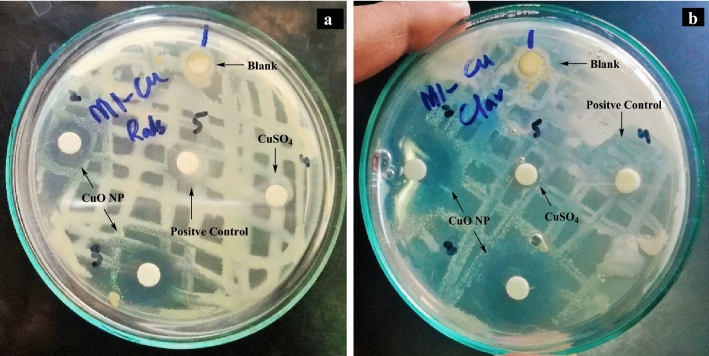
Zone of inhibition of (**a**) *Ralstonia solanacearum* and (**b**) *Clavibacter michiganensis*.

The zone of inhibition of CuO NS (6.23%) against solanaceous wilt causing bacterial strains, namely, *Ralstonia solanacearum* and *Clavibacter michiganensis*, is shown in Fig. 6. It is visible that *Clavibacter michiganensis* was more affected than *Ralstonia solanacearum*. The synthesized CuO NS were tested compared to the aqueous extract of *Rhazya stricta*, the CuSO_4_ aqueous solution (6.23%), and known broad-spectrum antibiotic streptomycin sulfate (6.23%). The result is presented in Table 2 shows the biosynthesized CuO NS possessed higher antibacterial activity against both *R. solanacearum* and *C. michiganensis* with an inhibition zone measured as 17.30 mm and 17.97 mm, respectively (*p* < 0.05). Significantly improved antibacterial activity of the CuO NS against tested bacterial strains was recorded compared to standard antibiotics (*p* < 0.05).

**Table 2:** Antibacterial activity of CuO NS in comparison to control experiments.

Treatments	Zone of inhibition (mean ± S.E)		Overall mean
	*Ralstonia solanacearum*	*Clavibacter michiganensis*	
Blank	00.00 ± 0.00a	01.18 ± 0.00a	0.593a
CuO NS	17.30 ± 0.40	17.97 ± 0.12b	17.63b
CuSO_4 (aqu)_	00.00 ± 0.00a	6.43 ± 0.12c	3.217a
Streptomycin sulfate	08.18 ± 0.31c	14.83 ± 0.20d	11.51c

Interestingly both selected bacteria started to develop resistance after 18 h against streptomycin, and clear hallow around disk became turbid after 24 h. In contrast, selected bacteria did not show any growth around CuO NS, and clear hallow remained persistent after 24 h (Fig. 6). The aqueous extract of *Rhazya stricta* (blank) showed no activity against both *R. solanacearum* and *C. michiganensis*. The dense growth of both bacterial isolates was seen (Fig. 6, blank) around the disk containing an aqueous extract of *R. stricta*. A reason could be that the extract contains organic compounds suitable for the growth of bacterial isolates. The aqueous CuSO_4_ solution did not affect *R. solanacearum,* while against *C. michiganensis,* it showed a 6.43 mm inhibition zone. Overall, *Clavibacter michiganensis* was more susceptible to all experimental conditions than *Ralstonia solanacearum*. Similarly, the mean observed effect (17.63 mm) of CuO NS on bacterial isolates was recorded higher than all other treatments, including positive control experiments.

### Photocatalytic potential of CuO NS

**Figure 7:**
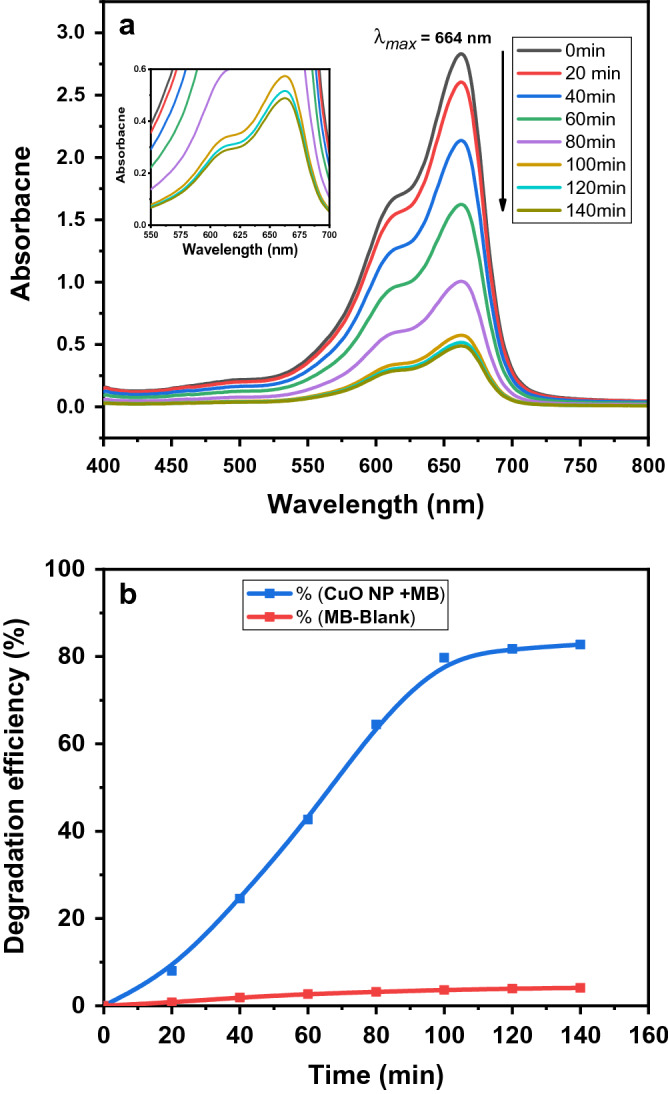
(**a**) Photocatalytic degradation of MB using CuO NS, (**b**) degradation efficiency (%) of MB. Both graphs were produced using OriginPro 2018 (64bit).

Methylene blue (MB) is a cationic aromatic dye used in the fabric industry. It has various harmful effects on humans, livestock, and aquatic organisms. Thus, it is necessary to remove MB and other hazardous dyes from industrial effluents. Metal oxide nanoparticles possess appreciable photocatalytic behavior against various coloring pigments^41^. The photocatalytic behavior of synthesized CuO NS against MB was examined under direct sunlight. An apparent time-dependent decrease in the absorbance band intensity of MB was observed after treatment with CuO NS. A blank degradation experiment showed merely 4.1% self-degradation of dye after 140 min. The catalysts (CuO NS) showed considerable degradation (82.7%) of MB after 140 min of reaction. Figure 7 clearly shows an incremental increase in the degradation efficiency till 100 min of reaction. After this time, degradation of MB became negligible, which could be due to the unavailable reactive sites of CuO NS. Mali and co-workers have reported up to 90% degradation of MB using Cu nanoparticles synthesized by *Celastrus paniculatus*^42^.

The mechanism for the photocatalytic degradation of synthesized CuO NS is summarized below

$${\text{CuO}}\;{\text{NP}} + {\text{h}}\nu + {\text{MB}}_{{{\text{aq}}}} \to {\text{CO}}_{{2}} + {\text{H}}_{{2}} {\text{O}} + {\text{by}}\;{\text{ products}}$$

At the initial stage, the CuO NS absorbs the photons from solar irradiation and moves to a photoexcited state. In semiconducting materials, electrons are excited to the conduction band upon irradiation, thereby producing electron–hole pairs.

$${\text{CuO}}\;{\text{NP}} + {\text{h}}\nu \to {\text{CuO}}e^{ - } + {\text{CuO}}h^{ + }$$

The valance band holes react with the hydroxyl ion (HO^−^) of the water (H_2_O) molecule to generate hydroxyl radicals (OH^•^) through oxidation. The *e*^*−*^ then reduces the Oxygen (O_2_) to form a superoxide ion (O_2_^•^^−^).

$${\text{CuO}}h^{ + } + {\text{ H}}_{{2}} {\text{O}} \to {\text{HO}}^{ \bullet }$$

$${\text{CuO}}e^{ - } + {\text{O}}_{{2}} \to {\text{O}}_{{2}}^{\bullet-}$$

These free radicals are highly reactive species causing oxidation of aromatic rings of MB dye to degrade into CO_2_, H_2_O and other degradation products.

$$ {\text{O}}_{{2}}^{\bullet-} + {\text{ HO}}^{ \bullet } + {\text{MB}} = {\text{degradation}}$$

**Figure 8:**
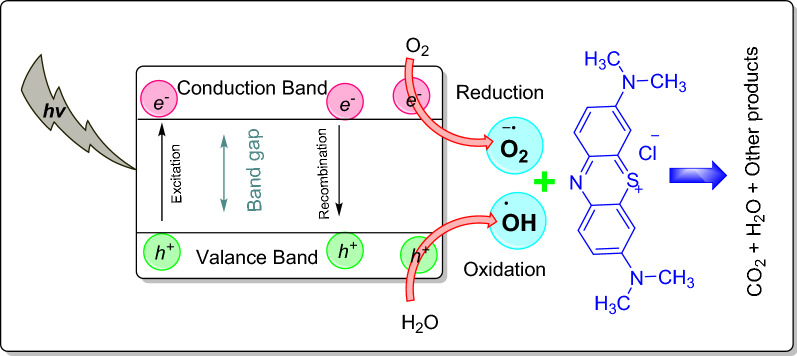
Mechanism of photocatalytic degradation of methylene blue using CuO NS. Image generated using ChemDraw professional v.17.

A detailed mechanism for the photocatalytic degradation of MB in the presence of CuO NS is presented in Fig. 8.

## Conclusions

The successful one-pot eco-friendly synthesis of Copper NS was achieved using an aqueous extract of *Rhazya stricta* in 24 h under mild conditions. Regular self-assembly of synthesized CuO NS was recorded in the form of three-dimensional cubical sheets with significant photocatalytic degradation efficiency against methylene blue. The nanosheets show a prominent bactericidal potential against wilt causing bacterial pathogens of solanaceous plants. The Cu liberated in the field as a by-product could also be used as a micronutrient by the crops.

## Data Availability

Data supporting this study's findings will be available from the corresponding author upon request.
