# Author Correction: Green synthesis of multifunctional carbon coated copper oxide nanosheets and their photocatalytic and antibacterial activities

**DOI:** 10.1038/s41598-021-96316-5

**Published:** 2021-08-13

**Authors:** Hamida Bibi, Mudassar Iqbal, Hassan Wahab, Mehmet Öztürk, Fei Ke, Zafar Iqbal, Muhammad Ishfaq Khan, Suliman Mohammed Alghanem

**Affiliations:** 1 Department of Soil and Environmental Sciences, The University of Agriculture, Peshawar, Pakistan; 2 Department of Agricultural Chemistry and Biochemistry, The University of Agriculture, Peshawar, Pakistan; 3 Physics Division, Pakistan Institute of Nuclear Science and Technology, Nilore, Islamabad, Pakistan; 4 Department of Chemistry, Faculty of Sciences, Muğla Sıtkı Koçman University, Muğla, Turkey; 5 Department of Applied Chemistry and State Key Laboratory of Tea Plant Biology and Utilization, Anhui Agricultural University, Hefei, People’s Republic of China; 6 Department of Weed Science and Botany, The University of Agriculture, Peshawar, Pakistan; 7 Biology Department, Faculty of Science, Tabuk University, Tabuk, Saudi Arabia

Correction to: *Scientific Reports* 10.1038/s41598-021-90207-5, published online 24 May 2021

In the original version of this Article, author Zafar Iqbal was incorrectly affiliated with “Department of Soil and Environmental Sciences, The University of Agriculture, Peshawar, Pakistan”. The correct affiliation is listed below.

Department of Agricultural Chemistry and Biochemistry, The University of Agriculture, Peshawar, Pakistan

The original Article has been corrected.

